# Integrated Analysis of RNA Binding Protein-Related lncRNA Prognostic Signature for Breast Cancer Patients

**DOI:** 10.3390/genes13020345

**Published:** 2022-02-14

**Authors:** Shaohua Xu, Jiahui Xie, Yanjie Zhou, Hui Liu, Yirong Wang, Zhaoyong Li

**Affiliations:** 1 Hunan Provincial Key Laboratory of Medical Virology, Institute of Pathogen Biology and Immunology, College of Biology, Hunan University, Changsha 410082, China; xushaohua2012@163.com (S.X.); xiejh@hnu.edu.cn (J.X.); 18373210670@163.com (Y.Z.); 15704925120@163.com (H.L.); 2 Bioinformatics Center, College of Biology, Hunan University, Changsha 410082, China; 3 Research Institute of Hunan University in Chongqing, Chongqing 401120, China

**Keywords:** breast cancer, RNA binding protein, long non-coding RNA, prognostic signature

## Abstract

Long non-coding RNAs (lncRNAs) have been well known for their multiple functions in the tumorigenesis, development, and prognosis of breast cancer (BC). Mechanistically, their production, function, or stability can be regulated by RNA binding proteins (RBPs), which were also involved in the carcinogenesis and progression of BC. However, the roles and clinical implications of RBP-related lncRNAs in BC remain largely unknown. Therefore, we herein aim to construct a prognostic signature with RBP-relevant lncRNAs for the prognostic evaluation of BC patients. Firstly, based on the RNA sequencing data of female BC patients from The Cancer Genome Atlas (TCGA) database, we screened out 377 differentially expressed lncRNAs related to RBPs. The univariate, least absolute shrinkage and selection operator (LASSO), and multivariate Cox regression analyses were then performed to establish a prognostic signature composed of 12-RBP-related lncRNAs. Furthermore, we divided the BC patients into high- and low-risk groups by the prognostic signature and found the overall survival (OS) of patients in the high-risk group was significantly shorter than that of the low-risk group. Moreover, the 12-lncRNA signature exhibited independence in evaluating the prognosis of BC patients. Additionally, a functional enrichment analysis revealed that the prognostic signature was associated with some cancer-relevant pathways, including cell cycle and immunity. In summary, our 12-lncRNA signature may provide a theoretical reference for the prognostic evaluation or clinical treatment of BC patients.

## 1. Introduction

Female breast cancer (BC) has become the leading cause of global cancer incidence in 2020, with approximately 2.3 million new cases and 685,000 deaths [1]. Metastasis and local or distant recurrence of tumors are the leading causes of mortality for BC patients [2]. BC is a highly heterogeneous tumor on the molecular level. Through the PAM50 classification system based on a 50-gene expression signature, current clinical practice has usually classified BC into five subtypes, including luminal A, luminal B, HER2-enriched, normal-like, and basal-like (triple-negative BC) [3]. However, as there is a heterogeneity of treatment response within the same subtype [2], the BC subtype still needs more exploration to obtain a higher therapeutic effect. Therefore, there is an urgent requirement to discover more prognostic signatures, which can stratify female BC patients to improve the prognosis of high-risk patients and minimize the overtreatment of low-risk patients.

RNA binding proteins (RBPs) are proteins that are highly conserved in evolution [4]. Through their RNA-binding domains, RBPs can directly bind with coding or non-coding RNAs and regulate their metabolic process, including RNA splicing, polyadenylation, localization, translation, and degradation [5,6]. Consequently, abnormal expression and function of RBPs, which are usually observed in various cancers, lead to the promotion or inhibition of tumorigenesis and progression [6]. Long non-coding RNAs (lncRNAs) are a type of non-coding RNAs with a length of more than 200 nucleotides. They are broadly involved in the regulation of gene expression by modulating chromatin architecture, transcription, mRNA stability, translation, and post-translational modifications [7]. In breast cancer, lncRNAs extensively affect cancer cell proliferation, metastasis, apoptosis, metabolism, immune evasion, and drug resistance [8]. Furthermore, the aberrant expression of lncRNAs endows them to predict the prognosis of breast cancer [9,10].

Accumulating studies have demonstrated RBPs regulate lncRNA generation at the transcriptional or post-transcriptional level in various cancers. As an RNA binding protein, the heterogeneous nuclear ribonucleoprotein E1 (hnRNP E1) binds to a nucleic acid structural element within exon 12 of PNUTS pre-RNA. It suppresses the splicing of the lncRNA-PNUTS isoform, which regulates epithelial-mesenchymal transition (EMT) and tumor progression through interaction with miR-205 as a competitive sponge [11]. Human antigen R (HuR), another RBP whose function has been extensively explored, not only physically interacts with and stabilizes lncRNAs, such as NEAT1 [12] and lncRNA-HGBC [13], but also recruits let-7-Ago2 complex to lncRNAs and promotes their degradation, including lncRNA-p21 [14] and HOTAIR [15]. Through the recruitment of the CCR4-NOT deadenylase complex, a critical component of the cytoplasmic RNA decay machinery, IGF2BP1 promotes degradation of HULC [16], and Tristetraprolin (TTP) may promote decay of HOTAIR [17,18], respectively. However, lncRNA NEAT1 is destabilized by AUF1 [19] and PABPN1 [20] but stabilized by SPSF1 [21] through different mechanisms. Interestingly, one recent genome-wide analysis of lncRNA half-lives in humans revealed that RBP-lncRNA interactions mainly enhance the stability of lncRNA with only one exon [22]. Additionally, N6-methyladenosine (m6A) writers and erasers, as well as readers, are all RBPs that modulate m6A modification and function of lncRNAs [23], primarily by affecting their stability and changing their expression levels [24,25,26]. Recently, RBP has also been reported to regulate the transcription of lncRNA. For example, ZC3H4, a CCCH zinc finger domain-containing RBP, occupies broad promoter regions and represses non-coding transcription events in human cells [27]. With WDR82 that binds Ser5-phosphorylated RNA Pol II, ZC3H4 forms a complex that suppresses lncRNAs transcribed from enhancers and promoters by transcription termination, which is activated by the inefficiently spliced first exon of lncRNAs [28]. Collectively, RBPs can function as regulatory proteins and control the expression of lncRNAs in multiple ways. 

Although autophagy, aerobic glycolysis, stemness, and immune-related lncRNA prognostic signatures have been developed for the prognosis evaluation of breast cancer [29,30,31,32], the RBPs-related lncRNA predictive risk model for breast cancer remains unknown. Therefore, our study aimed to develop a valuable prognostic signature based on RBP-related lncRNAs for female BC patients. We have systematically identified lncRNAs that were correlated with the expression of RBPs in BC. With 12-RBP-related lncRNAs, we constructed a prognostic signature, which can serve as an independent prognostic biomarker for BC patients. Furthermore, we found the prognostic signature was tightly connected with immune checkpoints and tumor mutational burden (TMB). In total, our study developed an RBP-related lncRNA signature to predict the prognosis of female BC patients and provided a theoretical basis for the treatment of BC.

## 2. Materials and Methods

### 2.1. Acquisition and Processing of Data

The transcriptome profiling, simple nucleotide variations, and the corresponding clinical information of BC patients were downloaded from The Cancer Genome Atlas (TCGA) database (https://portal.gdc.cancer.gov, accessed on 7 November 2020). Additionally, the disease-specific survival (DSS) information of TCGA-BRCA (breast invasive carcinoma) was downloaded from the University of California, Santa Cruz (UCSC) Xena database (http://xena.ucsc.edu/, accessed on 1 November 2021). Then, we also obtained the PAM50 subtype information of TCGA-BRCA [33]. The protein expression data of BC patients were collected from the Clinical Proteomic Tumor Analysis Consortium (CPTAC) database (https://cptac-data-portal.georgetown.edu/study-summary/S015, accessed on 2 August 2021). Since there are rare male breast cancer patients in the TCGA database, we kept breast cancer samples only from female patients (1096 tumor tissues and 112 normal tissues). Patients with overall survival (OS) < 30 days were excluded to ensure the accuracy of the prognostic analysis in our study. Furthermore, the 842 cases with complete clinical information were retained for further analysis.

### 2.2. Identification of Differential Expressed RBP-Related lncRNAs in BC Tissues

Differentially expressed genes were calculated by R package DESeq2 (P.adj < 0.05) [34]. The differentially expressed proteins between BC tumor and normal tissues were identified by the *t*-test and Benjamini–Hochberg method (FDR < 0.05). In addition, we also collected 2283 RBP genes from the previous study [35]. Differentially expressed RBP genes were strictly identified with consistent protein and mRNA expression data. The Pearson correlation coefficient |R| > 0 and *p*-value < 0.05 were considered as significant. The lncRNAs with |log2 (fold change)| > 1 and P.adjust < 0.05 were identified as differentially expressed lncRNAs. To filter low-expressed lncRNAs, we retained differential expression lncRNAs that were expressed in at least 80% of the tumor samples. The correlation between RBP genes and differentially expressed lncRNAs was evaluated by the Pearson correlation analysis, and the RBP-related lncRNAs were identified according to the standard of the |R| > 0.4 and the *p*-value < 0.001.

### 2.3. Construction of the RBP-Related Prognostic Signature

The 842 cases with complete clinical information were used as the entire cohort. With the R package caret [36], all cases were randomly assigned into the training (*n* = 590) and validation dataset (*n* = 252) at a ratio of 7:3 [37]. The differences in clinical features of the patients between these two datasets were analyzed by the Fisher’s exact test. The prognostic lncRNAs associated with RBP were identified based on a univariate Cox regression analysis for OS with the R package survival in the training dataset [38]. Then, some tightly correlated lncRNAs of them were deleted by the least absolute shrinkage and selection operator (LASSO) to avoid over-fitting of the model by using the R package glmnet [39]. Then, the RBP-related lncRNA signature was constructed through the multivariate Cox regression analysis according to the lowest Akaike information criterion (AIC) value. Additionally, the risk score was calculated as the following formula: Risk score = Σ(Exp_i_ ∗ Coef_i_). The Coef represents the coefficients of each lncRNA, and the Exp represents the expression level of each lncRNA.

### 2.4. Assessment and Validation for the Prognostic Value of the RBP-Related lncRNA Signature

The training dataset was categorized into high- and low-risk groups based on the median risk score. Similarly, the validation dataset and entire cohort were also classified into high- and low-risk groups based on the median risk score of the training dataset, respectively. Subsequently, the Kaplan–Meier survival analysis and log-rank test were used to assess the difference in OS or DSS between the high- and low-risk groups. The time-dependent receiver operating characteristic (ROC) curves at 1-, 3-, and 5-year OS were performed by the R package survivalROC (survivalROC package version v.1.0.3, https://cran.r-project.org/web/packages/survivalROC/index.html, accessed on 22 December 2020).

### 2.5. Clinical Correlation Analysis for Risk Score

We firstly performed the Kaplan–Meier survival analysis and log-rank test to evaluate the differences in OS between the high- and low-risk groups in different subgroups stratified by several clinical features based on the entire cohort, including age (≥60 years old, <60 years old), TNM stage (stage I–II, stage III–IV), T stage (T1–2, T3–4), N stage (N0, N1–3), M stage (M0), and PMA50 subtypes (luminal A, normal-like, basal-like, luminal B, and HER2-enriched). As the number of patients in the M1 stage was only 15, the survival analysis was not performed. Furthermore, the univariate Cox, multivariate Cox, and ROC curve analyses for OS were used to assess the independent prognostic value of the RBP-related lncRNA signature based on the risk score and above clinical features.

### 2.6. Functional Enrichment Analysis

Functional annotation of RBP genes related to the lncRNAs in the prognostic signature was performed by R package ClusterProfiler [40]. The terms of Gene Ontology (GO) or Kyoto Encyclopedia of Genes and Genomes (KEGG) with P.adjust < 0.05 were considered to be significantly enriched. Additionally, the gene set enrichment analysis (GSEA) between the high- and low-risk groups in the entire cohort was executed based on the KEGG pathway (c2.cp.kegg.v7.4.symbols.gmt) with the GSEA software (v.4.1.0; http://www.broadinstitute.org/gsea/index.jsp, accessed on 18 March 2021). The pathways with the normalized enrichment score |NES| > 1 and FDR < 0.05 were taken to be significantly enriched.

### 2.7. Correlation between the Risk Score and Tumor Mutational Burden

The tumor mutational burden (TMB) was defined as the total number of gene mutations per million bases. Only 3 types of mutations were considered in our analysis, including single-nucleotide polymorphism (SNP) and insertion-deletion (InDel) mutations. The TMB value of each patient was calculated by using the Perl programming language. Moreover, we divided BC patients with risk scores into high- and low-TMB groups according to the optimal cut-point of the TMB determined by the surv_cutpoint function of the survminer package (survminer package version v.0.4.8, https://rpkgs.datanovia.com/survminer/index.html, accessed on 6 November 2020). The correlation between the risk score and TMB was evaluated by the Spearman correlation analysis, and the difference in TMB between high- and low-risk groups was calculated with the Wilcoxon test. 

### 2.8. Statistical Analysis

All the analyses were performed in the R software (version v.4.0.2, https://www.r-project.org/, accessed on 22 June 2020). The DESeq2 and *t*-test were used to identify differentially expressed genes and proteins between the two different groups, respectively. The Pearson or Spearman methods were used to perform a correlation analysis. Fisher’s exact test was used to evaluate the difference of categorical variables in two different groups. The differences in numerical variables between two different groups were calculated with the Wilcoxon test. Statistical significance was set at *p* < 0.05 unless otherwise stated.

## 3. Results

### 3.1. Identification of Differentially Expressed lncRNAs Associated with RBPs in Female BC Patients

**Figure 1:**
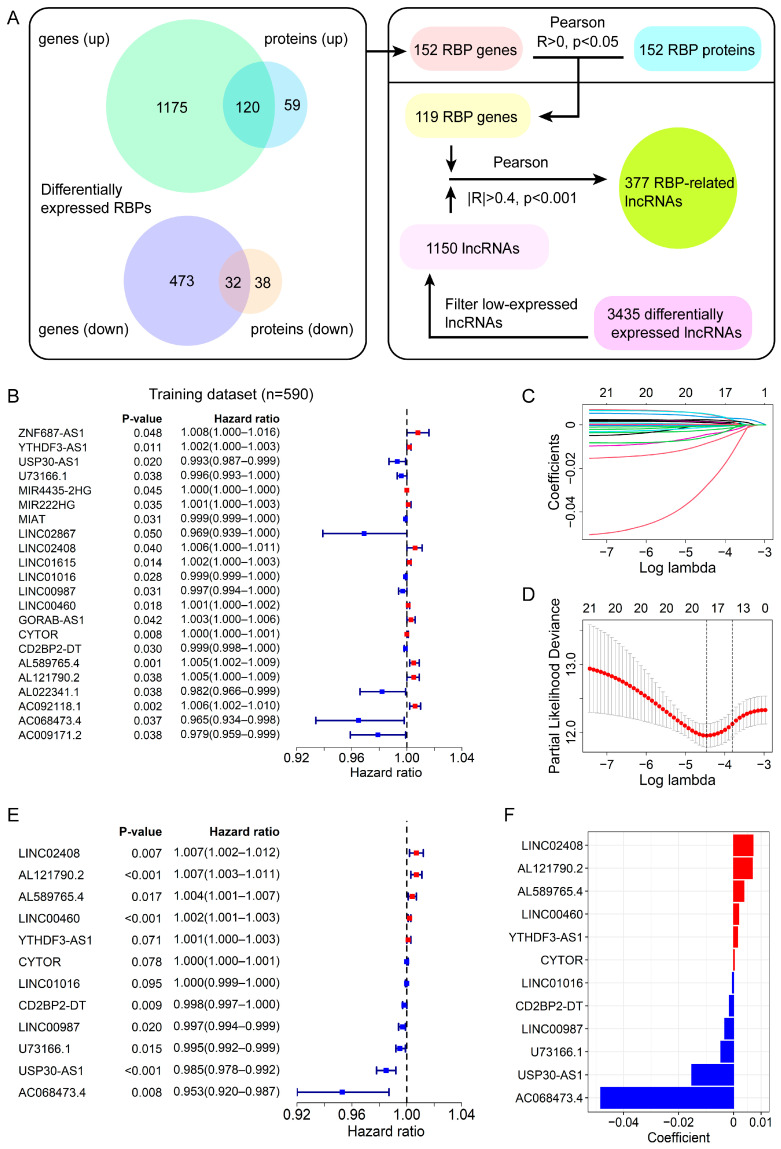
Identification of differential expressed lncRNAs related to RBPs and establishment of a 12-lncRNA prognostic signature. (**A**) The flowchart for identification of RBP-related lncRNAs. (**B**) Forest plot of univariate Cox regression analysis for the 22 RBP-related lncRNAs correlated with the OS of BC patients in the training dataset. (**C**) The LASSO coefficient profiles of the 22 prognosis-associated lncRNAs. The upper abscissa represents the number of lncRNAs with non-zero coefficients under the corresponding lambda. (**D**) The cross-validation graph shows the optimal parameter selection with minimum criteria in the LASSO model. The first black dashed line shows the best parameter (lambda). The upper abscissa represents the number of lncRNAs with non-zero coefficients under the corresponding lambda. (**E**) Forest plot of the multivariate Cox regression analysis for the 12 RBP-related lncRNAs. (**F**) The coefficients of the 12 lncRNAs from multivariate Cox regression analysis.

The differentially expressed lncRNAs associated with RBP in BC are identified as in Figure 1A. We gathered a total of 1800 differentially expressed RBP genes by comparing 1096 BC samples with 112 normal samples from the TCGA database. Then, 249 differentially expressed RBPs were identified by comparing 103 BC samples and three normal samples from the CPTAC database. By taking the intersection of transcriptome profiling and proteomic datasets, we obtained 152 RBP genes. Finally, 119 RBP genes were screened out by positive statistical significance. Therefore, here we used the mRNA expression level of these RBP genes to represent their protein expression for subsequent analysis. We also filtered out 1150 differentially expressed lncRNAs widely expressed in tumors. Subsequently, 377 RBP-related lncRNAs were identified based on the Pearson correlation coefficient |R| > 0.4 and *p*-value < 0.001.

### 3.2. Development of the RBP-Associated lncRNA Signature

The entire cohort containing 842 BC cases was randomly assigned into the training dataset (*n* = 590) and the validation dataset (*n* = 252). The clinical features are presented in Table S1, which showed the clinical characteristics of patients in these two datasets were similar (*p*-value > 0.05). We identified the 22 lncRNAs significantly associated with OS from 377 RBP-related lncRNAs by a univariate Cox regression analysis in the training dataset (Figure 1B). These significant lncRNAs were subjected to a LASSO Cox regression analysis for avoiding the overfitting of the model, and 18 candidate lncRNAs were selected for further analysis (Figure 1C,D). Finally, 12 lncRNAs for constructing the prognostic signature were screened out via a multivariate Cox regression analysis (Figure 1E). The specific description and coefficients of these lncRNAs are displayed in Table 1 and Figure 1F.

### 3.3. Evaluation and Validation for the Prognostic Ability of the RBP-Related lncRNA Signature

**Figure 2:**
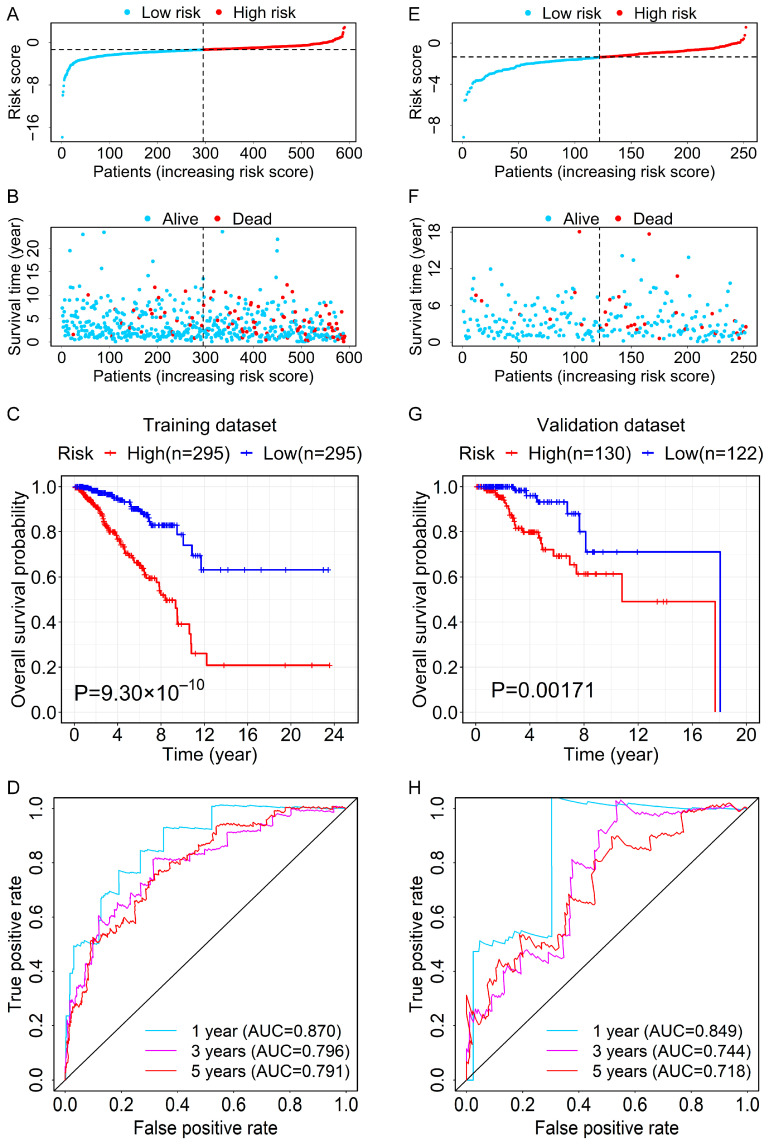
Evaluation and verification for the prognostic value of the RBP-related lncRNA signature. (**A**) Risk scores for BC patients of the high- and low-risk groups in the training dataset. (**B**) The scatterplot of overall survival time and status of BC patients in the high- and low-risk groups from the training dataset. (**C**) The OS curve for BC patients in the high- and low-risk groups of the training dataset. (**D**) The time-dependent ROC curves at 1-, 3-, and 5-year OS of the 12-lncRNA prognostic signature in the training dataset. (**E**) Risk scores for BC patients of the high- and low-risk groups in the validation dataset. (**F**) The scatterplot of overall survival time and status of BC patients in the high- and low-risk groups from the validation dataset. (**G**) The OS curve for BC patients in the high- and low-risk groups of the validation dataset. (**H**) The time-dependent ROC curves at 1-, 3-, and 5-year OS of the 12-lncRNA prognostic signature in the validation dataset.

Subsequently, the risk score of each case in the entire cohort was calculated with the coefficients and expression levels of these 12 lncRNAs. BC patients in the training dataset were categorized into high- and low-risk groups based on the median value of the risk score (risk score = −1.3618) (Figure 2A). The OS and survival status of patients between high- and low-risk groups are depicted in Figure 2B, which showed the high mortality in the high-risk group. Furthermore, we performed the Kaplan–Meier analysis and found that patients in the low-risk group had longer OS than that of the high-risk group (Figure 2C). The time-dependent ROC curves were used for evaluating the prediction efficiency of the prognostic signature. Furthermore, the area under the curve (AUC) values for 1-, 3-, and 5-year OS reached 0.870, 0.796, and 0.791, respectively (Figure 2D).

To test the prognostic capability of the RBP-related lncRNA signature, we used the median risk score of the training dataset (risk score = −1.3618) to divide the validation dataset and the entire cohort into the high- and low-risk groups, respectively. The distributions of risk score and survival status of patients between high- and low-risk groups in the validation dataset are displayed in Figure 2E,F. The result of the overall survival analysis suggested that patients in the high-risk group had a poorer prognosis than those in the low-risk group (Figure 2G). The AUC values of ROC curves at 1-, 3-, and 5-year OS were 0.849, 0.744, and 0.718, respectively (Figure 2H). The distribution diagrams of risk score and survival status of the entire cohort are shown in Figure S1A,B. The heatmap for the expression levels of the 12-RBP-related lncRNAs for each patient is presented in Figure S1C. Patients in the high-risk group had a shorter OS (Figure S1D). The ROC curves for risk score demonstrated that the AUC values for 1-, 3-, and 5-year OS were 0.867, 0.785, and 0.772, respectively (Figure S1E).

Besides the OS, we also analyzed the differences in DSS of high- and low-risk groups in all three datasets. Overall, the low-risk group indicated a higher survival probability of patients (Figure S2A–C). Moreover, the AUC values of the 1-, 3-, and 5-year ROC curves for risk score were also greater than 0.7 in all circumstances (Figure S2D–F). These results confirmed that the 12-RBP-related lncRNA prognostic signature could predict the survival outcomes of BC patients.

To further test the prognostic power of the 12-RBP-related lncRNA signature, we set a signature based on the 10 most differentially expressed genes (DEGs). Most of the coefficients of the top 10 DEGs based on the multivariable Cox regression analysis were not significant (Table S2). When we classified BC patients into high- and low-risk groups with the same method, the result of the overall survival analysis was not significantly different between the two groups in the validation dataset (Figure S3). Furthermore, the AUC values were much lower than those of the 12-RBP-related lncRNA signature.

### 3.4. Relationship between the Prognostic Signature and Clinical Features

**Figure 3:**
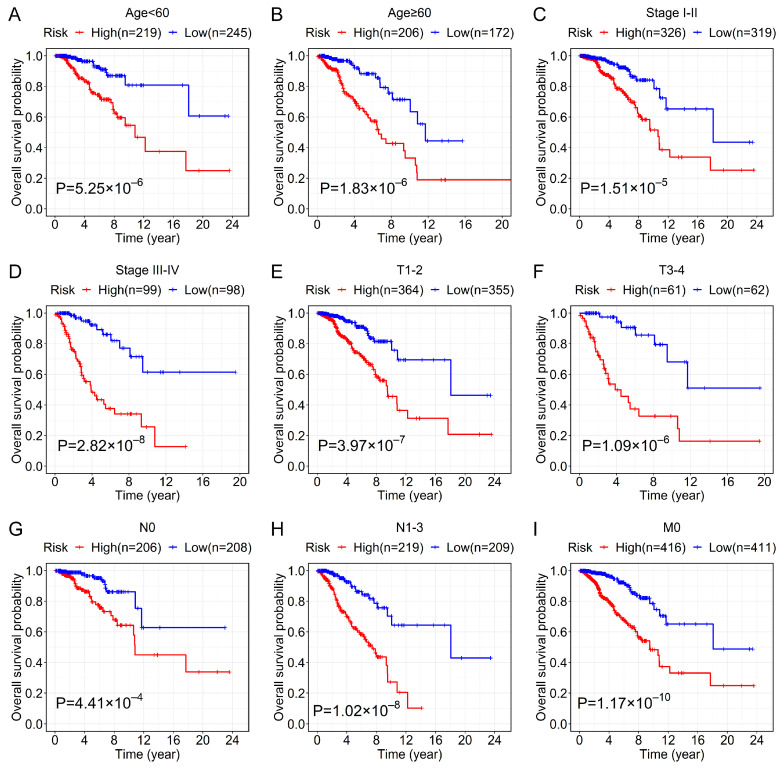
Kaplan–Meier survival analyses for BC patients of the high- and low-risk groups in the different clinical subgroups. The OS curve for BC patients of the high- and low-risk groups in the different subgroups stratified by clinical features based on the entire cohort, including age < 60 years old (**A**), age ≥ 60 years old (**B**), stage I–II (**C**), stage III–IV (**D**), T1–2 (**E**), T3–4 (**F**), N0 (**G**), N1–3 (**H**), and M0 (**I**).

To assess the broad applicability for prognosis of the 12-lncRNA signature, we performed the stratification survival analysis to confirm its prognostic ability in various subgroups of the 842 cases in the entire cohort. The OS of patients in the low-risk group was higher than that of the high-risk group in the subgroups classified by age, TNM stage, T stage, N stage, and M stage (Figure 3). As for the PAM50 subtype, we found that patients in the high-risk groups had a poorer prognosis in these subtypes, including luminal A, normal-like, and basal-like (Figure S4A–C). However, there was no significant difference in survival analysis between high- and low-risk groups in the remaining subtypes, like luminal B and HER2-enriched (Figure S4D,E).

**Figure 4:**
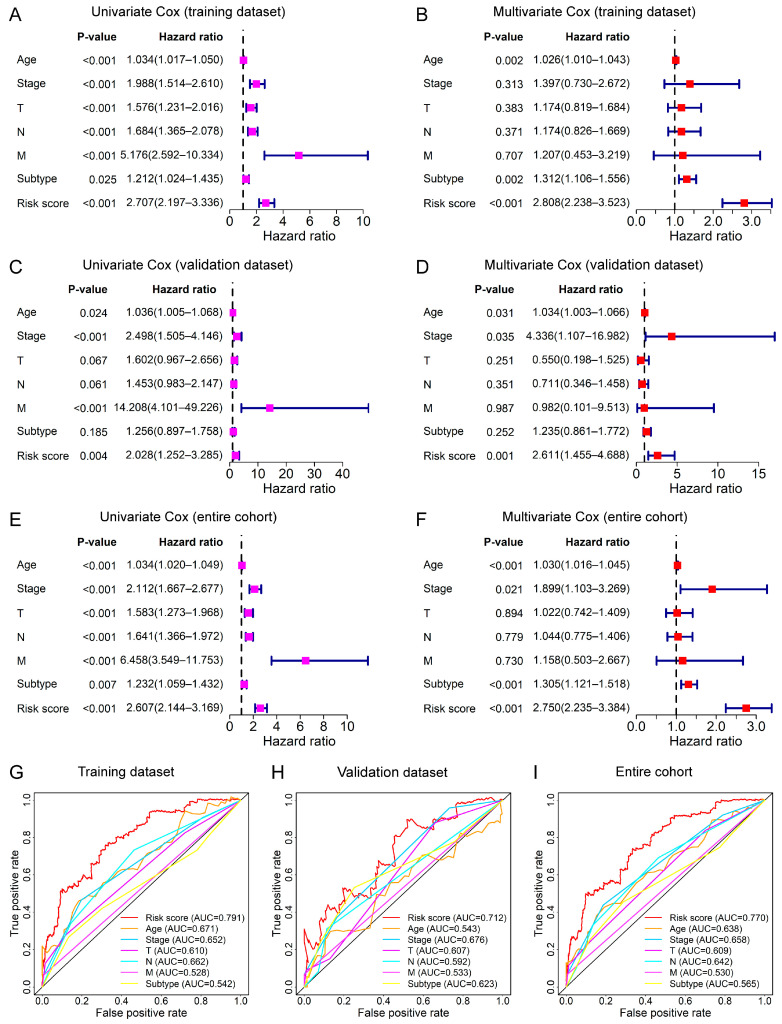
Assessment of the lncRNA signature as an independent prognostic factor for OS. Forest plots of the univariate (**left**) and multivariate (**right**) Cox regression analyses of risk score and several clinical features based on the OS in the training dataset (**A**,**B**), validation dataset (**C**,**D**), and entire cohort (**E**,**F**). The ROC curves at 5-year OS of the risk score and clinical features in the training dataset (**G**), validation dataset (**H**), and entire cohort (**I**).

Cox regression analyses for OS were conducted to confirm the risk score of the 12-lncRNA signature can be regarded as an independent indicator of prognosis for BC patients. In the training dataset, the univariate Cox analysis showed that the risk score of the 12-lncRNA signature was associated with the prognosis of BC patients (Figure 4A), and the multivariate Cox analysis implied that the prognostic ability of the risk score was unrelated to clinical parameters, such as age, TNM stage, T stage, N stage, M stage, and PAM50 subtype (Figure 4B), indicating the risk score can be taken as an independent prognostic indicator for BC patients. Similarly, Cox regression analyses showed that the risk score was still an independent prognostic indicator for BC patients in the validation dataset and entire cohort (Figure 4C–F).

The AUC values at the 5-year OS of the risk score were also higher than those of other clinical parameters, such as age, stages, and subtype in all conditions (Figure 4G–I). These results suggested that the 12-lncRNA signature for BC patients had better performance in prognostic accuracy.

### 3.5. Construction of Co-Expression Network and Functional Enrichment Analysis for Exploring Biological Processes Relevant to the Prognostic Signature

**Figure 5:**
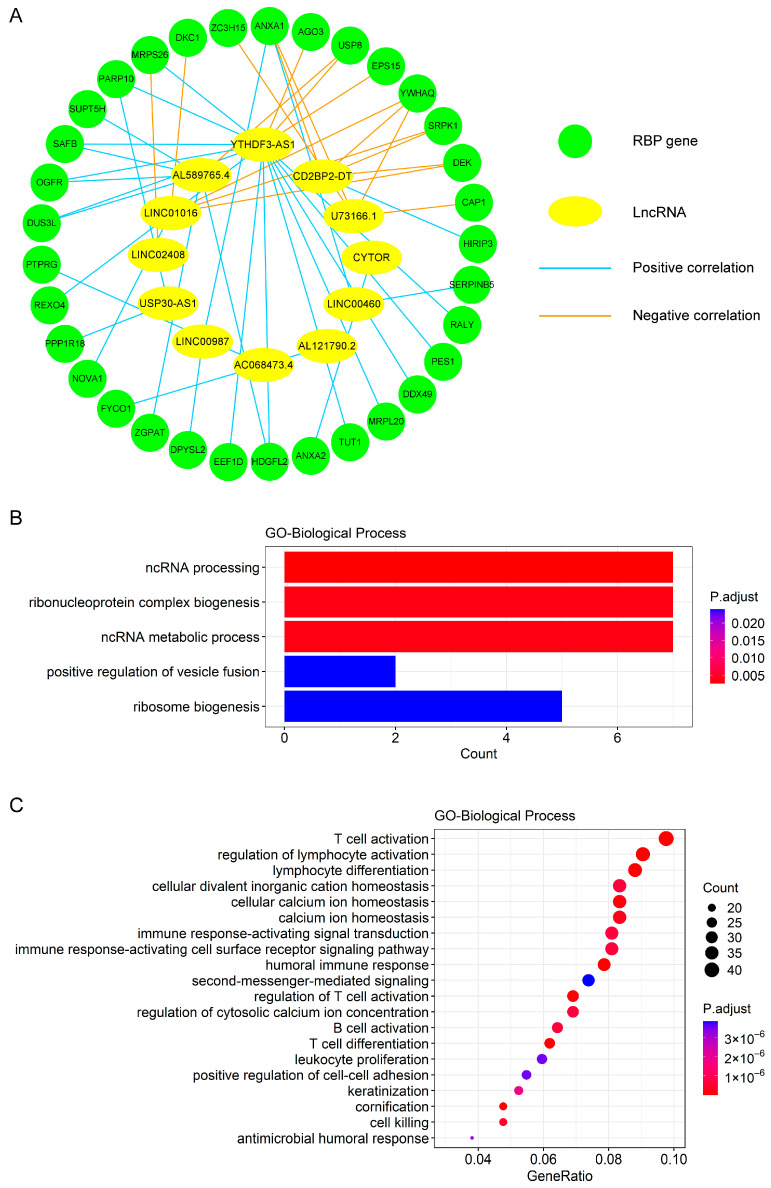
The co-expression network and GO enrichment analysis. (**A**) A co-expression network comprised of 12 lncRNAs in the prognostic signature and 33 RBP genes correlated to them. (**B**) The top 5 GO biological processes with the significant enrichment of the 33 RBP genes. (**C**) The top 20 GO biological processes with the significant enrichment of the differentially expressed genes between the high- and low-risk groups in the entire cohort.

To explore the biological process relevant to the 12-lncRNA signature, we constructed the co-expression network of these lncRNAs and 33 related RBPs according to the correlation analysis result (Figure 5A and Table S3). In line with expectations, the 33 RBP genes were majorly enriched in some ncRNA-related processes, including ncRNA processing and the ncRNA metabolic process (Figure 5B). To further test the physical association between these lncRNAs and RBPs, we performed lncRNA–RBP interactions in silico by the lncPro [41], which can predict the lncRNA–protein interaction based on the sequence analysis and generate an interaction score. A score over 50 indicates the possibility of an interaction between the lncRNA and protein. We found the majority of these lncRNAs (11 of 12) could potentially be bound by at least one co-expressed RBP (Table S4), further supporting the connection of biological function between the lncRNA signature and RBPs.

We also investigated which biological processes were associated with the risk score to reveal the possible mechanisms that affected the prognosis of BC patients. In total, 480 differentially expressed genes were identified between the high- and low-risk groups in the entire cohort. A GO enrichment analysis revealed that these genes were primarily enriched in some biological processes related to immunity, such as T cell differentiation, regulation of lymphocyte activation, and lymphocyte differentiation. The top 20 terms are shown in Figure 5C. The KEGG pathway enrichment analysis also demonstrated that these differentially expressed genes were mainly enriched in some pathways associated with immunity, such as cytokine–cytokine receptor interaction, natural killer cell mediated cytotoxicity, and the chemokine signaling pathway (Figure S5A). 

Moreover, the GSEA analysis was used to further explore the pathways associated with the prognostic signature. We discovered that some immune-related pathways were enriched in the low-risk group (Figure S5B), which suggested that the tumor tissues in the low-risk group may have a higher immune infiltration degree. Whereas several cancer-related pathways, like cell cycle and citrate cycle (TCA cycle) pathways, were enriched in the high-risk groups (Figure S5C). Interestingly, we uncovered that ubiquitin-mediated proteolysis, RNA degradation, and basal transcription factors pathways, which may be regulated by lncRNAs, were also enriched in the high-risk group (Figure S5C).

### 3.6. Association between the Prognostic Signature and Immune Checkpoint Genes or TMB

**Figure 6:**
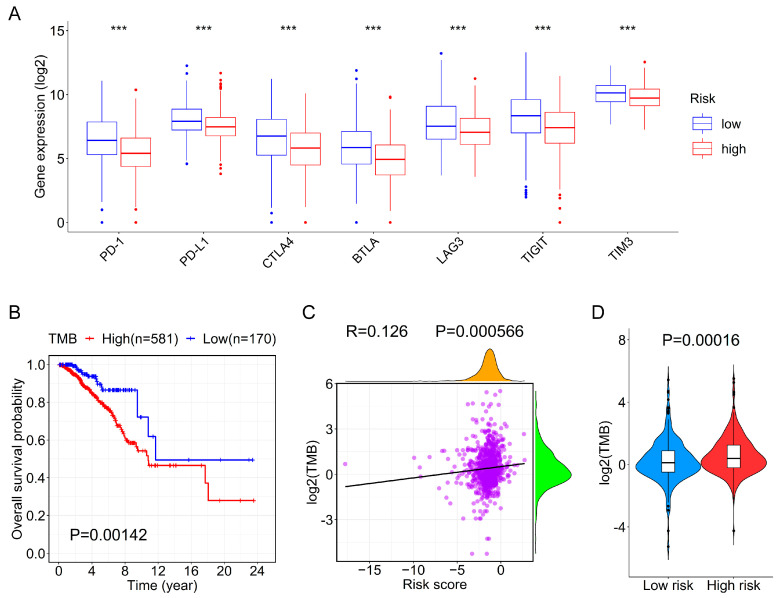
The correlation between the risk score and immune checkpoint genes or TMB. (**A**) The box plot shows the expression levels of some representative immune checkpoint genes between the high- and low-risk groups in the entire cohort. (**B**) The OS curve for BC patients in the high- and low-TMB groups based on the mutation data. (**C**) The correlation analysis between TMB and risk score. (**D**) The violin plot shows the difference in TMB between the high- and low-risk groups. *** P.adjust < 0.001.

To further elucidate the relationship between the 12-lncRNA signature and immunity, we compared the expression levels of the 48 immune checkpoint genes [42] between high- and low-risk groups. Interestingly, we found that except for 11 genes whose expression changes were not significant, the remaining 37 genes were significantly up-regulated in the low-risk group (P.adjust < 0.05) (Table S5). Compared with the high-risk group, some representative immune checkpoint genes, including *PD-1*, *PD-L1*, *CTLA4*, *BTLA*, *LAG3*, *TIGIT*, and *TIM3*, were significantly increased in the low-risk group (Figure 6A).

Since TMB is highly related to tumor immunogenicity, we also investigated the relationship between risk score and TMB. The Kaplan–Meier survival curve implicated that TMB was associated with the poor prognosis of BC patients (Figure 6B). Furthermore, we found that TMB was significantly positively correlated with risk score (Figure 6C) and the TMB values in the high-risk groups were also significantly higher than those in the low-risk group (Figure 6D), which may explain the worse prognosis of patients of high-risk groups.

## 4. Discussion

BC is a family of tumors with variable molecular features and responses to therapy [3,43]. However, the traditional clinicopathological prognostic variables and the few molecular prognostic markers are insufficient to reflect the biological and clinical heterogeneity of BC [43]. Thus, the exploration of potential prognostic biomarkers is urgently required for the guidance of the individualized treatment and management of BC patients. As a kind of protein that can function via interaction with target RNAs, multiple RBPs have been found to be dysregulated and relevant to the prognosis of BC patients [44,45,46]. Furthermore, the prognostic signature constructed by three RBPs has also been established in BC [47]. Although the expression of lncRNAs has been widely regulated by RBPs at the transcription or post-transcription level [11,12,13,14,15,16,17,18,19,20,21,27,28], the prognostic value of lncRNAs associated with RBPs in BC remains largely unknown. Consequently, we herein addressed this by constructing a prognostic signature for BC patients based on the RBP-related lncRNAs. 

In this study, we initially identified 377 RBP-related lncRNAs in female BC patients based on the RNA-seq and proteomic data (Figure 1A). Next, we divided the entire cohort into a training dataset and a validation dataset. Subsequently, through the univariate, LASSO, and multivariate Cox regression analyses in the training dataset, we screened out 12-RBP-related lncRNAs that were related to the prognosis of BC patients. Based on the expression of these 12 lncRNAs in the training dataset, we constructed a prognostic signature and calculated the risk score. Furthermore, we evaluated the signature in the training dataset and verified it in the validation dataset and entire cohort. Our analyses showed the risk score was associated with a worse prognosis of patients and could be taken as an independent prognostic indicator. Moreover, the survival analyses in various subgroups stratified by clinical features (age, stages, and PAM50 subtype) revealed the signature had broad applicability. Additionally, the AUC value of the ROC curves for risk score was greater than that of other clinical features, indicating the 12-lncRNA signature had higher accuracy.

Among these 12 lncRNAs constructing the prognostic signature, LINC02408, AL589765.4, AL121790.2, YTHDF3-AS1, LINC00460, and CYTOR functioned as risk factors for the prognosis of BC patients, while AC068473.4, USP30-AS1, U73166.1, LINC00987, CD2BP2-DT, and LINC01016 acted as protective factors. The previous studies have uncovered that LINC00460 is a strong risk marker of BC [48] and can promote breast cancer progression by sponging miR-489-5p [49]. Similarly, CYTOR (also known as LINC00152) can increase cell proliferation, migration, and invasion of BC and is related to the bad outcome of BC patients [50,51]. Furthermore, in accordance with our findings, some other studies mainly based on the bioinformatics analysis have also reported that LINC02408 [52] and YTHDF3-AS1 [53] are connected with the poor prognosis of BC patients, but USP30-AS1 [54], U73166.1 [55], and LINC01016 [30] can be taken as the protective factors of prognosis. Therefore, as the above evidence supported, the 12 screened RBP-related lncRNAs may be tightly connected with the prognosis of BC patients. 

Through the Pearson correlation analysis, we found 33 RBP genes were tightly connected with the 12 lncRNAs in the prognostic signature. Notably, some of these RBPs have been reported to affect the development and prognosis of BC. For example, PES1 can promote BC growth by differentially regulating the transcriptional activity and protein stability of ERα and ERβ, as well as the expression of their target genes [56]. Another RBP, ANXA1, which promotes the progression of BC by facilitating the Treg cell-mediated anti-tumor immunity, is associated with the poor survival of BC patients and a higher risk of breast malignancy [57,58]. Additionally, SRPK1 is essential for the invasion and metastasis of BC [59], and DKC1 is relevant to the unfavorable clinical features and worse prognosis of BC patients [60]. However, high CAP1 expression may predict a good clinical outcome in BC [61]. Furthermore, we found 33 RBP genes were closely associated with the ncRNA processing and metabolic process (Figure 5B), indicating their potential ability in influencing the abundance of lncRNAs. For instance, as one of these RBPs, SAFB (also known as SAFB1) has multiple binding sites across the lncRNA MALAT1, and SAFB knockdown significantly increased the expression level of MALAT1 [62]. Taken together, 33 RBPs might function in breast cancer progression by regulating the generation or process of the 12 lncRNAs in the signature.

To further understand biological processes and potential mechanisms that lead to differences in the survival of patients in the high- and low-risk groups, we performed the GO and KEGG enrichment analyses, as well as the GSEA analysis. The results showed many immune-related pathways were enriched in the low-risk groups (Figure 5C and Figure S5A,B). Therefore, we speculated that patients in the low-risk group exhibited an active immune status. On the contrary, the result of the GSEA analysis showed the cell cycle and citrate cycle (TCA cycle) pathways were enriched in the high-risk groups (Figure S5C). The above findings may help to explain a better prognosis of patients in the low-risk group than that of patients in the high-risk group.

Targeting immune checkpoints can provide new insights for the treatment of cancer, and immune checkpoint inhibitors, such as atezolizumab and nab-paclitaxel, have been approved for the first-line therapy of PD-L1–positive metastatic triple-negative BC [63]. Besides, increasing studies also demonstrated the role of some other novel immune checkpoint inhibitors in the treatment of BC [64]. Thus, we deeply analyzed the expression of 48 immune checkpoint genes in the high- and low-risk groups and found the expression levels of 37 immune checkpoint genes were significantly higher in the low-risk group. These findings indicate patients in the low-risk groups may benefit from immune checkpoint therapy. Consistent with one previous study [65], the BC patients with high TMB had shorter survival (Figure 6B). Furthermore, we also discovered the TMB values were positively correlated with the risk scores of BC patients (Figure 6C,D), which testified the prognostic value of our 12-lncRNA signature, at least in part. Additionally, previously conducted studies have elucidated cancer cells with higher TMB are more likely to generate potent immunogenic neoantigens, which leads to T cell priming and improves the chance of an effective host immune treatment response [66]. Therefore, other immunotherapy methods other than immune checkpoint treatment may be more suitable for the BC patients in the high-risk group.

In summary, our study identified and validated a reliable prognostic signature based on the 12-RBP-related lncRNAs, which possesses independent prognostic significance for female BC patients. Meanwhile, we also discovered the prognostic signature was related to the expression of immune checkpoint genes and TMB. Thus, this signature may be utilized to improve the prognosis of BC patients. Although our study was based on a large sample of multi-omics data, there were certain limitations. The BC cases used in our research were only derived from the public databases (retrospective cohort). Therefore, further validation in the prospective cohort can validate the prognostic accuracy of the signature. Additionally, the carryout of function and mechanism studies for these 12 lncRNAs can further support their clinical applications. Nonetheless, the 12-lncRNA signature in our study can potentially be used as a risk marker to assess the prognosis of BC patients.

## 5. Conclusions

We established an RBP-related lncRNA signature for evaluating the prognosis of female BC patients. The prognostic signature, verified for accuracy, wide-scale applicability, and independence, was associated with tumor immunity. Therefore, this lncRNA signature could serve as a promising prognostic biomarker, which may provide the theoretical foundation for personalized prognostic management and individualized therapies of BC patients.

## Figures and Tables

**Table 1:** The coefficients of 12 RBP-related lncRNAs based on the multivariable Cox regression analysis in the training dataset.

Gene Symbol	Ensembl ID	Genomic Location	Coefficient
LINC02408	ENSG00000203585	Chr12:67,443,105–67,590,771	0.007062056
AL121790.2	ENSG00000259087	Chr14:37,556,158–37,567,095	0.00683141
AL589765.4	ENSG00000249602	Chr1:151,763,384–151,769,501	0.003727625
LINC00460	ENSG00000233532	Chr13:106,374,477–106,384,315	0.001867941
YTHDF3-AS1	ENSG00000270673	Chr8:63,167,725–63,168,442	0.00146521
CYTOR	ENSG00000222041	Chr2:87,454,781–87,636,740	0.000252682
LINC01016	ENSG00000249346	Chr6:33,867,506–33,896,914	−0.000429308
CD2BP2-DT	ENSG00000260219	Chr16:30,354,665–30,357,116	−0.001513116
LINC00987	ENSG00000237248	Chr12:9,240,003–9,257,960	−0.003223765
U73166.1	ENSG00000230454	Chr3:50,260,303–50,263,358	−0.004631155
USP30-AS1	ENSG00000256262	Chr12:109,052,349–109,053,984	−0.015207547
AC068473.4	ENSG00000267409	Chr18:79,610,747–79,612,303	−0.048280801

Note: the reference genome version used for the genomic location was GRCh38. Chr: chromosome.

## Data Availability

The data analyzed in this study are available in the TCGA database (https://portal.gdc.cancer.gov, accessed on 7 November 2020), CPTAC database (https://cptac-data-portal.georgetown.edu/study-summary/S015, accessed on 2 August 2021), and UCSC Xena database (http://xena.ucsc.edu/, accessed on 1 November 2021).

## Supplementary Materials

The following supporting information can be downloaded at: https://www.mdpi.com/article/10.3390/genes13020345/s1, Table S1. The clinical features of breast cancer patients. Table S2. The coefficients of the top 10 DEGs based on the multivariable Cox regression analysis. Table S3. The 33 RBP genes correlated with the 12 lncRNAs in the prognostic signature. Table S4. The interaction score of the 12 lncRNAs and 33 RBPs yielded by the lncPro. Table S5. The results of differential expression of the 48 immune checkpoint genes between the high- and low-risk groups. Figure S1. Verification for the prognostic value of the RBP-related lncRNA signature in the entire cohort. Figure S2. Evaluation and verification of the RBP-related lncRNA prognostic signature for predicting DSS of BC patients. Figure S3. Evaluation and verification for the prognostic value of the top 10 DEGs signature. Figure S4. Kaplan–Meier survival curves for BC patients of the high- and low-risk groups in the different PAM50 subtypes. Figure S5. Gene enrichment analysis for KEGG pathways between the high- and low-risk groups in the entire cohort.
