# Supplementary material for: Integrated Analysis of RNA Binding Protein-Related lncRNA Prognostic Signature for Breast Cancer Patients
Source: Genes (Basel). 2022 Feb 14;13(2):345. doi: 10.3390/genes13020345 (PMC8872055; doi:10.3390/genes13020345)
Supplement: Supplementary file 1 [file genes-13-00345-s001.zip › genes-1574947-supplementary.pdf]

› **Supplementary Materials**

Table S1. The clinical features of breast cancer patients.

**Characteristics**	**Training dataset (*n*=590)**		**Validation dataset (*n*=252)**		**Entire cohort (*n*=842)**		**P-value**
	n	**%**	***n***	**%**	***n***	**%**	
Age(year)							0.705
<60	328	55.59%	136	64.68%	464	55.11%	
⩾60	262	44.41%	116	35.32%	378	44.89%	
TNM stage							0.291
I	101	17.12%	57	22.62%	158	18.76%	
II	345	58.48%	142	56.35%	487	57.84%	
III	133	22.54%	49	19.44%	182	21.62%	
IV	11	1.86%	4	1.59%	15	1.78%	
T stage							0.717
T1	156	26.44%	76	30.16%	232	27.55%	
T2	345	58.48%	142	56.35%	487	57.84%	
T3	68	11.52%	27	10.71%	95	11.28%	
T4	21	3.56%	7	2.78%	28	3.33%	
N stage							0.158
N0	287	48.65%	127	50.40%	414	49.17%	
N1	197	33.39%	87	34.52%	284	33.73%	
N2	73	12.37%	19	7.54%	92	10.93%	
N3	33	5.59%	19	7.54%	52	6.17%	
M stage							1.00
M0	579	98.14%	248	98.41%	827	98.22%	
M1	11	1.86%	4	1.59%	15	1.78%	
Survival status							0.171
Deceased	95	16.10%	31	12.30%	126	14.96%	
Living	495	83.90%	221	87.70%	716	85.04%	

Table S2. The coefficients of the top 10 DEGs based on the multivariable Cox regression analysis.

Gene symbol	Coefficient	Hazard ratio	95% CI (Hazard ratio)	P-value
NEK2	0.00002	1.00002	0.99987-1.00017	0.77872
MMP11	0.00001	1.00001	1-1.00002	0.08126
COL10A1	-0.00002	0.99998	0.99992-1.00004	0.51371
LINC01614	0.00088	1.00088	1.00003-1.00174	0.04321
COL11A1	-0.00001	0.99999	0.99996-1.00003	0.64776
MYOM1	0.00056	1.00056	0.99832-1.00281	0.62288
GPAM	0.00005	1.00005	0.99993-1.00017	0.39856
FHL1	0.00001	1.00001	0.9999-1.00012	0.85389
TNS1	-0.00005	0.99995	0.99989-1.00001	0.09174
LYVE1	0.00034	1.00034	0.99999-1.0007	0.05997

Table S3. The 33 RBP genes that correlated with the 12 lncRNAs in the prognostic signature.

RBP gene	LncRNA	Correlation coefficient	P-value
USP8	YTHDF3-AS1	-0.409283787	1.64×10^{-45}
TUT1	YTHDF3-AS1	0.492422586	5.22×10^{-68}
SAFB	YTHDF3-AS1	0.41239633	3.02×10^{-46}
REXO4	YTHDF3-AS1	0.432797465	2.97×10^{-51}
RALY	YTHDF3-AS1	0.50204305	4.89×10^{-71}
PES1	YTHDF3-AS1	0.498890367	4.93×10^{-70}
PARP10	YTHDF3-AS1	0.447937049	3.37×10^{-55}
OGFR	YTHDF3-AS1	0.437641703	1.70×10^{-52}
MRPS26	YTHDF3-AS1	0.567350834	2.24×10^{-94}
MRPL20	YTHDF3-AS1	0.493937338	1.77×10^{-68}
HDGFL2	YTHDF3-AS1	0.515752019	1.60×10^{-75}
EPS15	YTHDF3-AS1	-0.442357922	1.01×10^{-53}
EEF1D	YTHDF3-AS1	0.540785304	2.92×10^{-84}
DUS3L	YTHDF3-AS1	0.423216991	7.36×10^{-49}
DDX49	YTHDF3-AS1	0.498814952	5.20×10^{-70}
AGO3	YTHDF3-AS1	-0.436466078	3.42×10^{-52}
PPP1R18	USP30-AS1	0.434960616	8.33×10^{-52}
PARP10	USP30-AS1	0.403074303	4.53×10^{-44}
ZC3H15	U73166.1	-0.410408031	8.92×10^{-46}
YWHAQ	U73166.1	-0.447877471	3.49×10^{-55}
CAP1	U73166.1	-0.48004093	2.98×10^{-64}
ANXA1	U73166.1	-0.432460495	3.61×10^{-51}
MRPS26	LINC02408	-0.405589696	1.19×10^{-44}
YWHAQ	LINC01016	-0.429217131	2.38×10^{-50}
SRPK1	LINC01016	-0.422991523	8.37×10^{-49}
NOVA1	LINC01016	0.442529048	9.11×10^{-54}

DKC1	LINC01016	-0.408121076	3.07×10^{-45}
DEK	LINC01016	-0.423470092	6.38×10^{-49}
DPYSL2	LINC00987	0.465343699	5.48×10^{-60}
ANXA1	LINC00987	0.422780586	9.43×10^{-49}
SERPINB5	LINC00460	0.471680434	8.43×10^{-62}
ANXA1	LINC00460	0.480668463	1.94×10^{-64}
ANXA2	CYTOR	0.477165584	2.11×10^{-63}
YWHAQ	CD2BP2-DT	-0.401867898	8.57×10^{-44}
SRPK1	CD2BP2-DT	-0.455597934	2.84×10^{-57}
HIRIP3	CD2BP2-DT	0.62704477	8.24×10^{-121}
DEK	CD2BP2-DT	-0.472604063	4.55×10^{-62}
ANXA1	CD2BP2-DT	-0.421148027	2.37×10^{-48}
ZGPAT	AL589765.4	0.462236845	4.11×10^{-59}
USP8	AL589765.4	-0.441702007	1.50×10^{-53}
SUPT5H	AL589765.4	0.404139304	2.58×10^{-44}
SAFB	AL589765.4	0.432394229	3.75×10^{-51}
OGFR	AL589765.4	0.41484807	7.88×10^{-47}
HDGFL2	AL589765.4	0.472093846	6.40×10^{-62}
DUS3L	AL589765.4	0.459386215	2.56×10^{-58}
FYCO1	AL121790.2	0.444202279	3.30×10^{-54}
PTPRG	AC068473.4	0.424008537	4.70×10^{-49}

Table S4. The interaction score of the 12 lncRNAs and 33 RBPs yielded by the lncPro.

RBP	LncRNA	Interaction score
PPP1R18	USP30-AS1	94.1676
ANXA2	CYTOR	90.5133
YWHAQ	LINC01016	90.0975
ANXA1	U73166.1	89.7058
MRPS26	LINC02408	89.5709
PARP10	USP30-AS1	86.6816
ANXA1	CD2BP2-DT	85.0979
ANXA1	LINC00460	79.8043
DEK	LINC01016	78.2294
SRPK1	LINC01016	78.2006
DKC1	LINC01016	77.9725
PARP10	YTHDF3-AS1	76.8786
USP8	AL589765.4	76.6731
HIRIP3	CD2BP2-DT	75.9459
ZC3H15	U73166.1	75.8884
SUPT5H	AL589765.4	75.5223
SAFB	YTHDF3-AS1	74.8977
OGFR	AL589765.4	74.3402

DUS3L	AL589765.4	74.3044
SAFB	AL589765.4	73.2533
HDGFL2	YTHDF3-AS1	72.3986
SRPK1	CD2BP2-DT	71.9792
DEK	CD2BP2-DT	71.2805
MRPS26	YTHDF3-AS1	71.1263
HDGFL2	AL589765.4	71.0666
ANXA1	LINC00987	69.3707
YWHAQ	CD2BP2-DT	67.2354
CAP1	U73166.1	67.0269
SERPINB5	LINC00460	66.6295
ZGPAT	AL589765.4	65.7774
DUS3L	YTHDF3-AS1	61.9488
REXO4	YTHDF3-AS1	61.099
FYCO1	AL121790.2	61.0164
MRPL20	YTHDF3-AS1	60.5053
NOVA1	LINC01016	59.4701
USP8	YTHDF3-AS1	58.6493
YWHAQ	U73166.1	57.7376
TUT1	YTHDF3-AS1	52.0828
EPS15	YTHDF3-AS1	51.0739
EEF1D	YTHDF3-AS1	48.8929
RALY	YTHDF3-AS1	43.7926
AGO3	YTHDF3-AS1	43.3126
PTPRG	AC068473.4	37.7127
DDX49	YTHDF3-AS1	34.1532
PES1	YTHDF3-AS1	28.2904
DPYSL2	LINC00987	28.2027
OGFR	YTHDF3-AS1	26.7317

Table S5. The results of differential expression of the 48 immune checkpoint genes in the high-and low-risk groups.

Gene name	Gene symbol	log2 (Fold Change)	P-value	P.adjust
CD27	CD27	-1.148709	1.07×10^{-30}	7.05×10^{-28}
CD48	CD48	-0.9565463	1.26×10^{-28}	5.38×10^{-26}
BTLA	BTLA	-1.269664	2.72×10^{-27}	7.74×10^{-25}
LAG3	LAG3	-1.124115	9.21×10^{-27}	2.41×10^{-24}
PD-1	PDCD1	-1.172503	1.04×10^{-26}	2.69×10^{-24}
TNFRSF14	TNFRSF14	-0.556548482	1.26×10^{-24}	2.19×10^{-22}
CD40LG	CD40LG	-1.074862	2.97×10^{-23}	4.09×10^{-21}
TIGIT	TIGIT	-1.031653035	6.17×10^{-21}	6.27×10^{-19}
PSGL-1	SELPLG	-0.59871339	1.51×10^{-20}	1.46×10^{-18}

TNFSF14	TNFSF14	-0.825576856	1.75×10^{-19}	1.44×10^{-17}
TMIGD2	TMIGD2	-1.038865107	4.20×10^{-19}	3.31×10^{-17}
CD28	CD28	-0.7268391	2.36×10^{-17}	1.46×10^{-15}
ICOS	ICOS	-0.9766838	2.94×10^{-17}	1.78×10^{-15}
CTLA4	CTLA4	-0.9253073	3.48×10^{-17}	2.09×10^{-15}
CD40	CD40	-0.5452667	1.15×10^{-16}	6.44×10^{-15}
CD200R1	CD200R1	-0.6736901	1.33×10^{-16}	7.35×10^{-15}
CD244	CD244	-0.7327014	1.02×10^{-15}	4.91×10^{-14}
IDO2	IDO2	-1.028678	1.33×10^{-15}	6.30×10^{-14}
VISTA	VSIR	-0.448487874	1.22×10^{-12}	3.40×10^{-11}
TNFRSF8	TNFRSF8	-0.604388969	4.46×10^{-12}	1.12×10^{-10}
TNFRSF25	TNFRSF25	-0.504007637	7.99×10^{-12}	1.90×10^{-10}
TNFRSF9	TNFRSF9	-0.682322953	1.08×10^{-11}	2.49×10^{-10}
OX40	TNFRSF4	-0.521509194	4.34×10^{-11}	8.89×10^{-10}
LAIR1	LAIR1	-0.403975	1.05×10^{-10}	1.98×10^{-09}
PD-L2	PDCD1LG2	-0.506019	1.07×10^{-10}	2.00×10^{-09}
PD-L1	CD274	-0.4919405	1.32×10^{-09}	1.93×10^{-08}
LGALS9	LGALS9	-0.4058531	7.13×10^{-09}	8.81×10^{-08}
CD200	CD200	-0.3236206	1.53×10^{-08}	1.75×10^{-07}
CD86	CD86	-0.3798495	1.66×10^{-08}	1.89×10^{-07}
KIR3DL1	KIR3DL1	-0.8648981	3.18×10^{-08}	3.40×10^{-07}
TIM3	HAVCR2	-0.2958509	5.57×10^{-07}	4.36×10^{-06}
CD70	CD70	-0.4917506	1.80×10^{-06}	1.24×10^{-05}
HHLA2	HHLA2	-0.6046469	8.60×10^{-06}	4.96×10^{-05}
TNFSF18	TNFSF18	-0.373244015	1.36×10^{-04}	5.67×10^{-04}
IDO1	IDO1	-0.509267	3.11×10^{-04}	1.17×10^{-03}
CD80	CD80	-0.2911305	7.46×10^{-04}	2.51×10^{-03}
CD160	CD160	-0.1927276	6.08×10^{-03}	1.54×10^{-02}
ADORA2A	ADORA2A	-0.2062096	3.63×10^{-02}	7.06×10^{-02}
TNFRSF18	TNFRSF18	-0.216934071	3.76×10^{-02}	7.28×10^{-02}
B7-H3	CD276	0.08529444	3.90×10^{-02}	7.51×10^{-02}
TNFSF15	TNFSF15	0.198038248	5.03×10^{-02}	9.29×10^{-02}
CD44	CD44	0.0876061	2.02×10^{-01}	2.94×10^{-01}
TNFSF9	TNFSF9	-0.097626131	2.81×10^{-01}	3.83×10^{-01}
BTNL2	BTNL2	-0.135373	3.43×10^{-01}	4.49×10^{-01}
NRP1	NRP1	-0.03387657	5.29×10^{-01}	6.30×10^{-01}
B7-H4	VTCN1	0.045778872	7.29×10^{-01}	8.00×10^{-01}
ICOSLG	ICOSLG	-0.02780938	7.81×10^{-01}	8.42×10^{-01}
TNFSF4	TNFSF4	-0.015090914	8.38×10^{-01}	8.85×10^{-01}

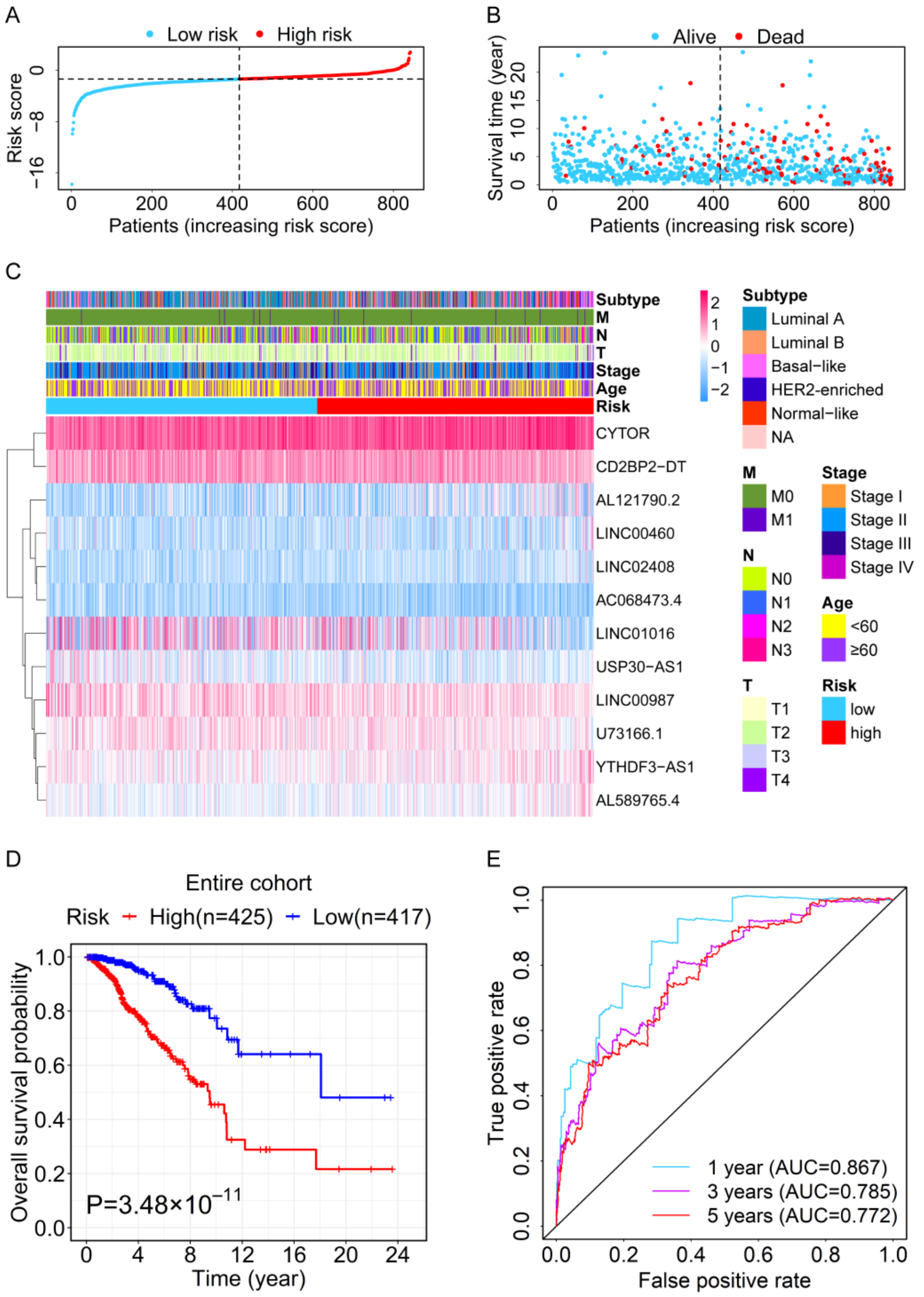

Figure S1. Verification for the prognostic value of the RBP-related lncRNA signature in the entire cohort

(A) Risk scores for BC patients of the high- and low-risk groups in the entire cohort. **(B)** The scatterplot of overall survival time and survival status of BC patients in the high- and low-risk groups from the entire cohort. **(C)** Heatmap of clustering analysis for the expression of 12 RBP-related lncRNAs between the high- and low-risk groups in the entire cohort. **(D)** The OS curve for BC patients in the high- and low-risk groups of the entire cohort. **(E)** The time-dependent ROC curves at 1-, 3-, and 5-year OS of the 12-lncRNA prognostic signature in the entire cohort.

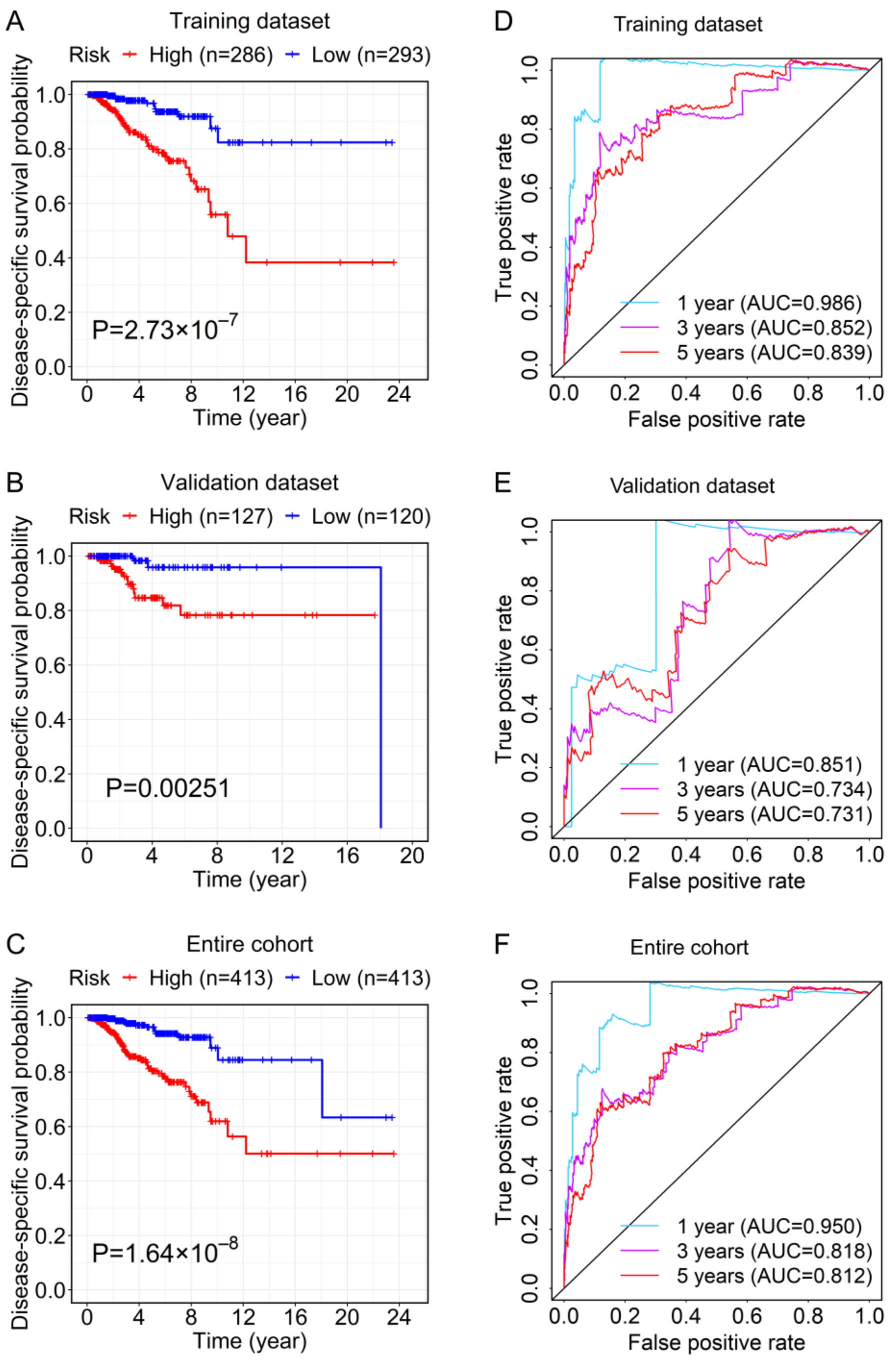

Figure S2. Evaluation and verification of the RBP-related lncRNA prognostic signature for predicting DSS of BC patients

(A) The DSS curve for BC patients in the high- and low-risk groups of the training dataset. **(B)** The DSS curve for BC patients in the high- and low-risk groups of the validation dataset. **(C)** The DSS curve for BC patients in the high- and low-risk groups of the entire cohort. **(D)** The time-dependent ROC curves at 1-, 3-, and 5-year DSS of the 12-lncRNA prognostic signature in the training dataset. **(E)** The time-dependent ROC curves at 1-, 3-, and 5-year DSS of the 12-lncRNA prognostic signature in the validation dataset. **(F)** The time-dependent ROC curves at 1-, 3-, and 5-year DSS of the 12-lncRNA prognostic signature in the entire cohort.

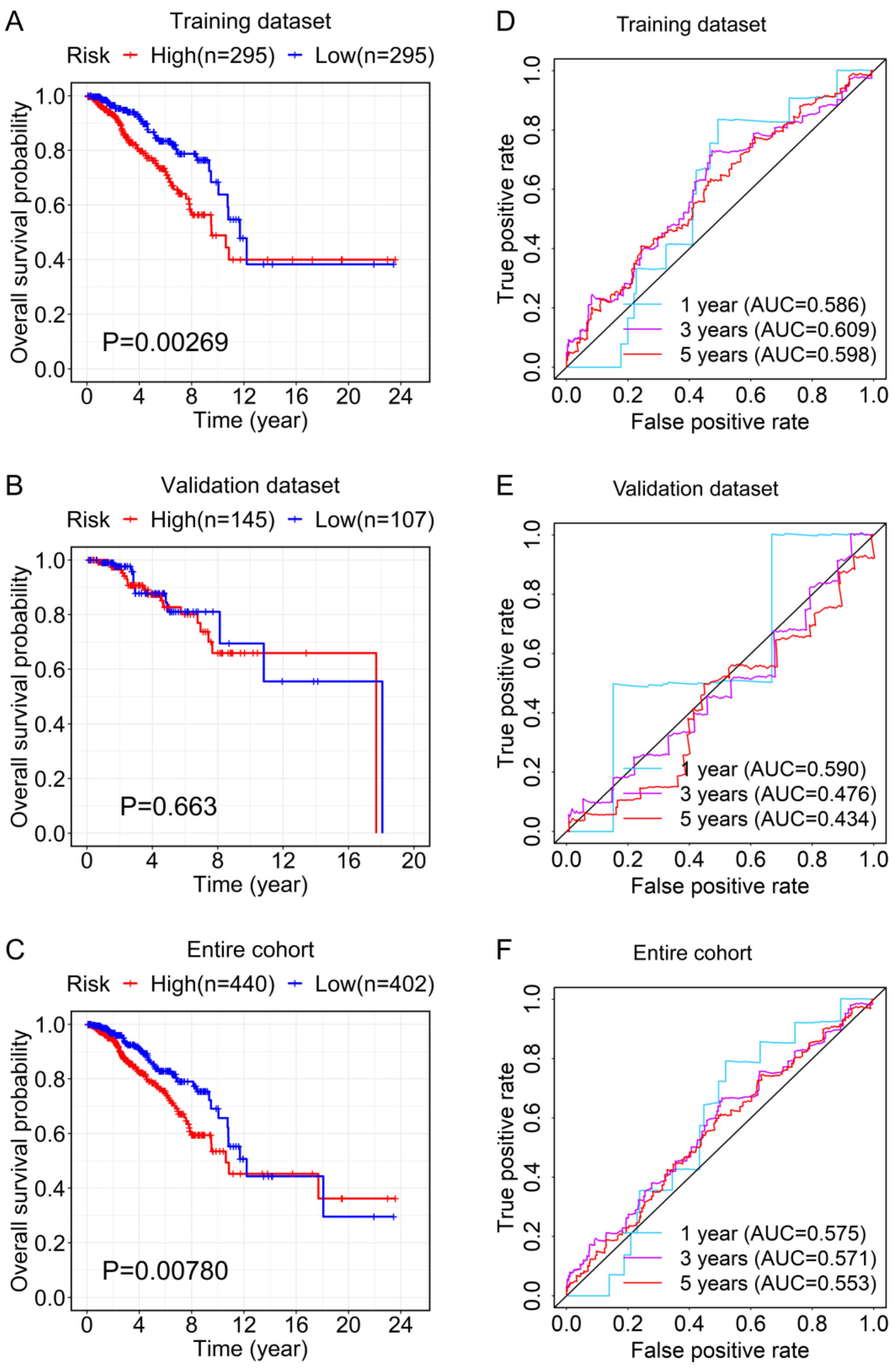

Figure S3. Evaluation and verification for the prognostic value of the top 10 DEGs signature. **(A-C)** The OS curve for BC patients in the high- and low-risk groups of the **(A)** training dataset, **(B)** validation dataset, and **(C)** entire cohort; **(D-F)** The time-dependent ROC curves at 1-, 3-, and 5-year OS of the top 10 DEGs prognostic signature in the **(D)** training dataset, **(E)** validation dataset, and **(F)** entire cohort.

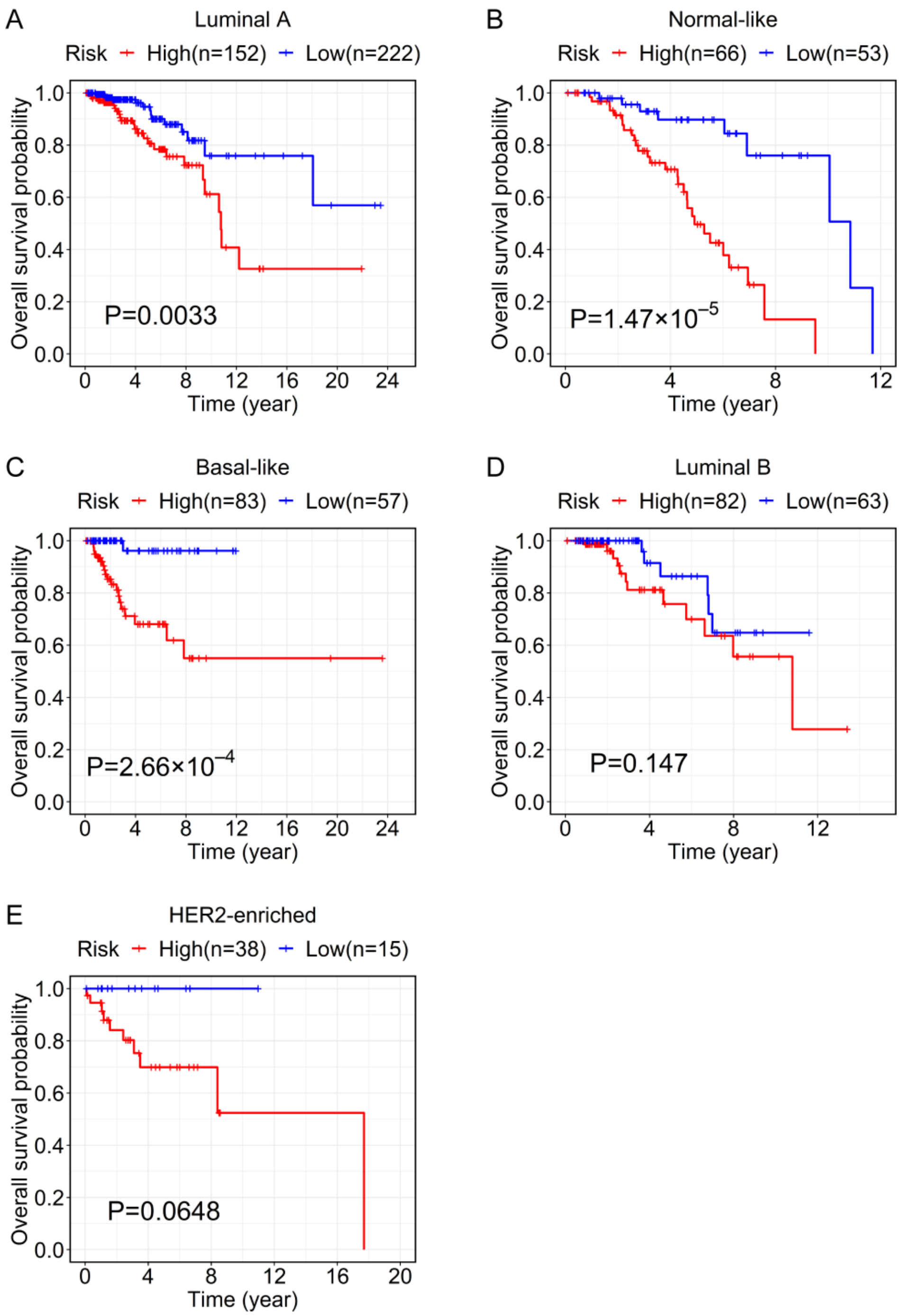

Figure S4. Kaplan-Meier survival curves for BC patients of the high- and low-risk groups in the different PAM50 subtypes

The OS curves for BC patients of the high- and low-risk groups in the different subgroups stratified by PAM50 subtypes based on the entire cohort, including luminal A **(A)**, normal-like **(B)**, basal-like **(C)**, luminal B **(D)**, and HER2-enriched **(E)**.

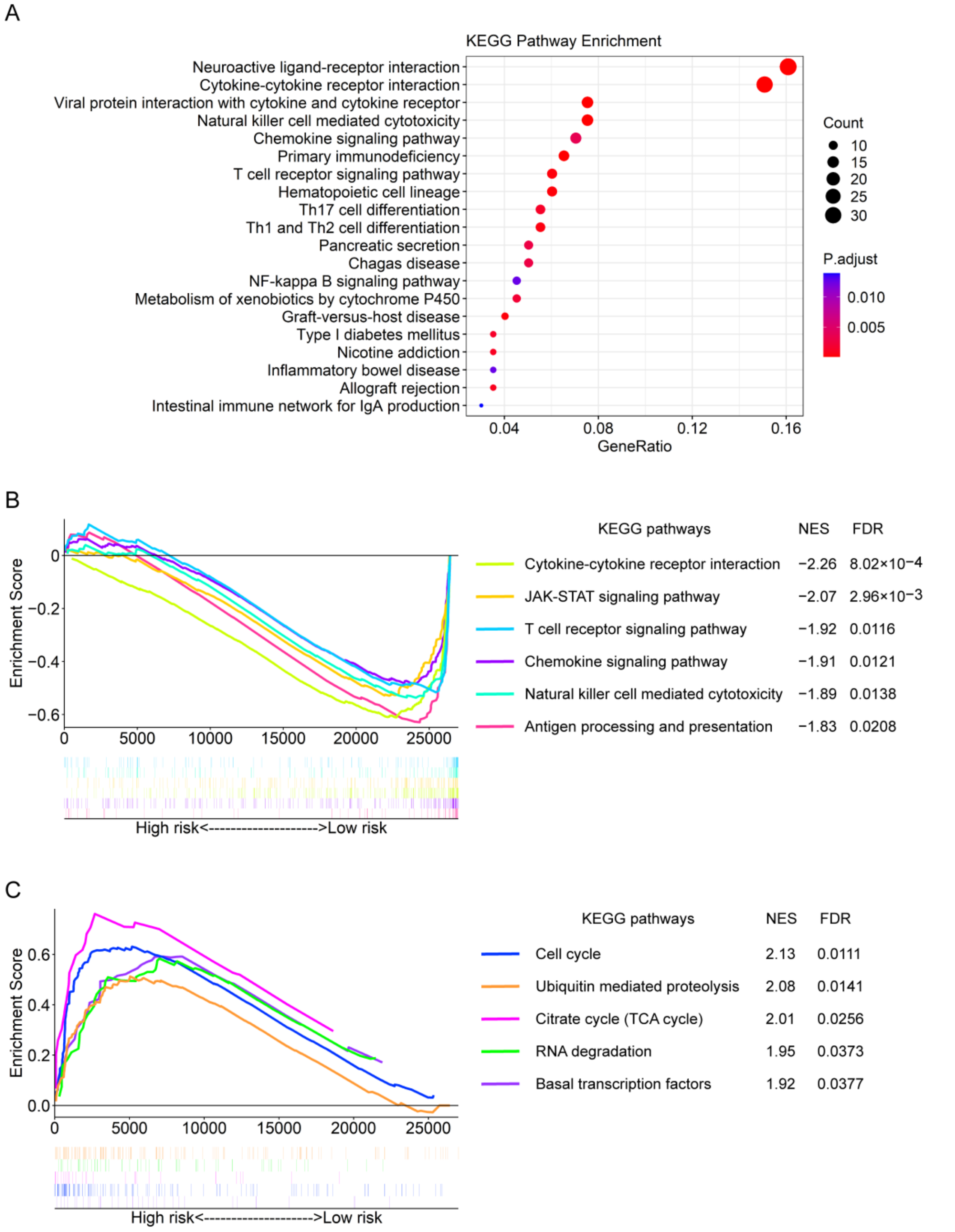

Figure S5. Gene enrichment analysis for KEGG pathways between the high- and low-risk groups in the entire cohort

(A) The top 20 KEGG pathways with the significant enrichment of the differentially expressed genes between the high- and low-risk groups in the entire cohort. **(B)** The KEGG pathways that were significantly enriched in the low-risk group by the GSEA analysis. **(C)** The KEGG pathways that were significantly enriched in the high-risk group by the GSEA analysis. NES: normalized enrichment score; FDR: false discovery rate.
